# Improvement of insulin resistance by Cyanidin 3-glucoside, anthocyanin from black beans through the up-regulation of GLUT4 gene expression

**DOI:** 10.1186/1753-6561-5-S8-P21

**Published:** 2011-11-22

**Authors:** Tetsuya Inaguma, Junkyu Han, Hiroko Isoda

**Affiliations:** 1 Graduate School of Life and Environmental Sciences University of Tsukuba, Tsukuba, Japan; 2 Alliance for Research on North Africa (ARENA) University of Tsukuba, Tsukuba, Japan

## Introduction

Black beans have been suggested to have a protective effect against obesity. Cyanidin 3-glucoside (Cy-3-G) belongs to the flavonoid class of molecules and is a member of the anthocyanin family that is present in black beans. Some studies indicated that Cy-3-G from black beans may be beneficial for the improvement of obesity and type Ⅱ diabetes. It has been reported that Cyanidin 3-glucoside ameliorates insulin sensitivity due by down-regulating the retinol binding protein 4 expression in diabetic mice [1]. Accumulation of neutral lipids, triglyceride (TG) in particular, is highly related to the development of insulin resistance and its consequences, such as type Ⅱ diabetes. Lipid droplet size regulation is central in the regulation of metabolism and in adipocytokines secretion such as TNF-α and adiponectin [2,3]. However, little is currently known about how Cy-3-G influences adipocyte differentiation and insulin resistance in 3T3-L1 adipocytes. The purpose of the present study was to determine the effects of Cy-3-G on GLUT4 gene expression to improve insulin resistance in 3T3-L1 adipocytes.

## Materials and methods

### Cell culture and treatment

Adipocytes, 3T3-L1 cells (Riken Cell Bank, Ibaraki, Japan), were maintained in Dulbecco’s modified Eagle’s medium (DMEM) (Sigma Chemical Co, St. Louis, MO, USA) supplemented with 10 % fetal bovine serum (FBS) (Biowest, Miami, FL, USA, Japan) and 1% 5,000 units/ml penicillin, 5,000 μg/mL streptomycin (PS) (Lonza Walkersville, MD,USA) and incubated in 37 °C in 5 % CO_2_. For adipocyte differentiation, 3T3-L1 cells were cultured at 3.0×10^5^ cells/well in 6-well plates. Cells were maintained until full confluence, which is often around 48h, prior to treatment, . And then, cells were treated with differentiation medium containing Dexamethazone (DEX) Solution, 3-Isobuthyl-1-Methylxanthine (IBMX) Solution, Insulin Solution, and Cy-3-G. DEX Solution, IBMX Solution, and Insulin Solution were purchased from Cayman Chemicals (Ann. Arbor, MI, USA). Cy-3-G derived from black soybean was purchased from Fujicco Co., Ltd. (Kobe, Japan). After 72 h of induction, medium was changed to DMEM containing insulin and Cy-3-G was added every 2 days. After 4 days of incubation from the initiation of differentiation, bioassays were performed.

### RNA extraction and real-time PCR

Total RNA was isolated from the 3T3-L1 cells using ISOGEN (Nippon Gene, Tokyo, Japan) according to the manufacturer’s instructions. The integrity of the RNA extracted from all samples was verified and quantified using NANO DROP 2000 (Agilent Technologies, CA). Total RNA was used for the single strand cDNA synthesis with a cDNA synthesis kit, SuperScript® III Reverse Transcriptase. Gene expression levels were analyzed by quantitative real-time PCR, using the Applied Biosystems 7500 FAST INSTURMENT (Applied Biosystems, Foster City, CA, U.S.A.). The oligonucleotide primers of mouse were obtained from Applied Biosystems (Foster City, CA, U.S.A.). The cDNA was denatured at 95 °C for 10 min, followed by 40 cycles of PCR (95 °C, 15 sec; 60 °C, 60 sec).

## Results and discussion

In Cy-3-G-treated cells, the number of droplets increased, while the lipid droplet size decreased. Cy-3-G significantly promoted the mRNA expressions of peroxisome proliferrator-activated receptor-γ (PPARγ) [4], CCAAT/enhancer binding protein α (C/EBPα) [4], and glucose transport 4 (GLUT4) [Table 1] in differentiated 3T3-L1 cells. PPARγ and C/EBPα mainly control adipocyte differentiation. GLUT4 takes in glucose on cell membrane. According to the results, Cy-3-G promotes adipocyte differentiation and uptake of glucose in a dose-dependent manner. In the present study, the concentrations of Cy-3-G treated were 20 μM, and 100 μM. Cy-3-G at 20 μM, and 100 μM significantly decreased TNF-α concentration and ROS production, and increased the adiponectin concentration in differentiated 3T3-L1 cell in a dose-dependent manner [4]. These effects suggest that Cy-3-G would contribute to the improvement of insulin resistance and its consequences. However, when we utilize black beans as food, the doses of Cy-3-G in black beans would be lower than the concentrations that we used in this study. Therefore, the study on the effects of Cy-3-G at lower concentrations, such as 1 μM, 5 μM and 10 μM, on adipocyte differentiation and adipocytokines secretion in differentiated 3T3-L1cells will be needed. Also, the anti-fat effects of Cy-3-G *in vivo* using diabetic model mice have not yet been widely reported. From our study, Cy-3-G derived from black bean as a food factor is expected to prevent life style-related disease.

**Table 1:** Effect of Cy-3-G on the gene expression of GLUT4 in 3T3-L1 adipocytes.

Sample	Relative GLUT4 gene expression level (% of Control)
Control	100 ± 1.5
Cy-3-G 20 µM	208.2 ± 1.4^*^
Cy-3-G 100 µM	226.57 ± 15.2^*^

Cy-3-G significantly induced the gene expression of GLUT4 in a dose-dependent manner. *p< 0.05 (vs Control)

## Conclusion

Cy-3-G has the potential to improve insulin resistance and its consequences in 3T3-L1 adipocytes through the up-regulation of GLUT4 gene expression. Additional study on insulin resistance using 3T3-L1and anti-fat effect using diabetic model mice will be needed to verify these results *in vivo*.
